# Three-Year Experience of Cytogenetic and Molecular Genetic Evaluation in Patients With Disorders of Sex Development at a Tertiary Care Centre in Eastern India

**DOI:** 10.7759/cureus.113844

**Published:** 2026-08-03

**Authors:** Sanjukta Sahoo, Bikasha Bihary Tripathy, Prabhas R Tripathy, Ravi K Narayan, KC Pradheep Kumar, Manisha R Gaikwad

**Affiliations:** 1 Anatomy, All India Institute of Medical Sciences, Bhubaneswar, Bhubaneswar, IND; 2 Pediatric Surgery, All India Institute of Medical Sciences, Bhubaneswar, Bhubaneswar, IND

**Keywords:** chromosomal analysis, cytogenetics, disorders of sex development, eastern india, molecular diagnostics

## Abstract

Objectives: Disorders of sex development (DSD) include a range of conditions in which chromosomal, gonadal, or anatomical sex deviates from the typical developmental pathway. The diagnostic approach to DSD has shifted from karyotyping to molecular genetic tools. This study evaluated the clinical presentation, cytogenetic spectrum, and diagnostic utility of conventional and advanced molecular genetic investigations in patients with suspected DSD managed at a tertiary care facility in Eastern India over a three-year period.

Methods: A retrospective observational study was conducted at the Genetics Laboratory of a tertiary care teaching hospital in Eastern India. Consecutive patients with clinically suspected DSD referred between January 2022 and December 2024 were included. Demographic and clinical data were obtained from referral records, and peripheral blood samples were analysed using standard G-banded karyotyping according to the International System for Human Cytogenomic Nomenclature (ISCN 2020). Fluorescence in situ hybridisation (FISH), chromosomal microarray analysis (CMA), and whole-genome sequencing (WGS) were selectively performed in cases with inconclusive cytogenetic findings, suspected structural chromosomal abnormalities, or complex phenotypes.

Results: A total of 98 suspected DSD cases were evaluated during the study period. The most frequent chromosomal constitution was 46,XY DSD (37, 37.8%), followed by 46,XX DSD (30, 30.6%) and sex chromosome DSD (21, 21.4%). Culture failure occurred in 10 (10.2%) samples. Advanced genetic techniques, including FISH, CMA, and WGS, improved diagnostic clarification in selected complex cases.

Conclusion: This experience highlights the importance of integrating contemporary high-resolution technologies with conventional cytogenetics to enhance the assessment, counselling, and treatment of individuals with DSD.

## Introduction

A variety of conditions that exist from birth and alter the typical process of sex determination and differentiation are referred to as disorders of sex development (DSD) [1,2]. Based on chromosomal makeup, the current classification system, which is based on the Chicago Consensus, separates DSD into three main categories: sex chromosome DSD, 46,XX DSD, and 46,XY DSD [3,4]. Although chromosomes are essential to this classification, sexual development is determined by a complex biological process influenced by structural, hormonal, and genetic factors [5]. The SRY gene on the Y chromosome is important for directing testicular development. This process influences both internal and external sexual differentiation [6]. Disruptions at any stage, whether in gonadal formation, hormone production, or receptor function, can lead to DSD [7]. Understanding of these processes has improved because of advances in genetic research, which have revealed the roles of different regulatory genes and pathways [8].

Estimating the prevalence of DSD remains difficult because of differences in reporting and diagnostic standards worldwide [9-12]. According to recent research, disruptions in male differentiation occur more frequently, making 46,XY DSD the most common category among various populations [13,14]. Data regarding DSD in Indian populations remain limited, and their interpretation is further complicated by regional differences in genetic diversity and consanguinity [15].

Accurate diagnosis is crucial for managing DSD. It influences gender assignment, estimation of gonadal function, assessment of cancer risk, and long-term care planning. The main method of investigation is G-banded karyotyping [16]. However, this method has limited ability to detect subtle or hidden genetic changes. The detection of mosaicism and copy-number variations (CNVs) is enhanced by molecular techniques such as fluorescence in situ hybridisation (FISH) and chromosomal microarray analysis (CMA) [17]. In complex cases, whole-genome sequencing (WGS) provides a clearer picture [17].

Given the need for better diagnosis, this study provides a brief overview of DSD cases assessed at a tertiary care facility in Eastern India. The importance of advanced techniques in shaping the diagnostic process is discussed in this context.

## Materials and methods

Study design and setting

This retrospective observational study was conducted in the Genetics Laboratory of the Department of Anatomy at a tertiary care institute in Eastern India. The centre has received referrals for suspected DSD for over 10 years. Its cytogenetics unit regularly works with the departments of paediatric surgery, endocrinology, and pathology. The study included all cases assessed between January 2022 and December 2024. Ethical approval was obtained before data retrieval, and the procedures adhered to the principles outlined in the Declaration of Helsinki.

Study population

A total of 98 individuals were referred for chromosomal evaluation because of clinical suspicion of DSD. Patients of any age or sex with clinically suspected DSD who underwent cytogenetic evaluation at our laboratory during the study period were included. Cases were excluded if clinical information was incomplete, an adequate biological sample for cytogenetic analysis was unavailable, or a definitive genetic diagnosis had already been established at another institution before referral. Samples with culture failure were retained for reporting laboratory performance and were analysed separately where appropriate. For each patient, basic details such as age, family history, and clinical features at presentation were recorded at referral.

Conventional cytogenetic study

Peripheral blood samples were processed according to recognised cytogenetic laboratory protocols. Lymphocyte cultures were established using RPMI-1640 medium supplemented with fetal bovine serum and stimulated with phytohaemagglutinin [14]. After a 72-hour incubation period, colcemid was introduced to arrest the cells in metaphase. Hypotonic treatment and fixation were performed, followed by G-banding using the trypsin-Giemsa method.

For interpretation, at least 20 metaphases were examined, and additional metaphases were assessed when mosaicism was suspected. Karyotypes were described according to the ISCN 2020 guidelines [18]. The unit maintains stringent internal quality checks, and difficult cases are jointly reviewed by two independent observers.

Advanced genetic techniques

Whenever chromosomal analysis alone did not fully explain the phenotype or structural rearrangements were suspected, additional tests were selectively applied: FISH for suspected mosaicism and structural abnormalities, CMA for genome-wide copy-number variation analysis, and WGS for a small number of highly complex cases.

The choice of advanced technique was based on clinical suspicion, the pattern of genital variation, developmental delay, or the results of imaging or hormonal studies. In a few cases, parental samples were also examined to clarify inheritance.

Clinical data compilation

Alongside laboratory testing, clinical teams summarised findings from physical assessment, hormonal profiles, imaging, and histopathology, where available. These elements were correlated with cytogenetic outcomes to support the final diagnosis and counselling decisions.

Statistical approach

This was a descriptive observational study; therefore, no hypothesis testing or comparative statistical analyses were performed. Data were entered into Microsoft Excel (Microsoft Corporation, Redmond, Washington) and analysed using IBM SPSS Statistics for Windows, Version 26 (Released 2018; IBM Corp., Armonk, New York). Continuous variables, such as age, were summarised using the mean ± standard deviation (SD), while categorical variables were presented as frequencies and percentages. The distributions of clinical presentations, cytogenetic findings, and molecular genetic investigations were described without inferential statistical testing, as the primary objective was to characterise the clinical and genetic spectrum of patients with DSD evaluated at our centre.

## Results

Demographic and clinical profile

A total of 98 patients with clinically suspected DSD were evaluated during the three-year study period. The mean age at presentation was 13.5 ± 12.3 years (range: birth to 45 years). Although patients ranged widely in age, 26 (26.5%) were referred during the first five years of life, including 8 (8.2%) in the neonatal period, reflecting early recognition of atypical genitalia. At the time of referral, 54 (55.1%) patients were initially assigned male sex and 44 (44.9%) female sex, resulting in a male-to-female ratio of 1.2:1. Following comprehensive clinical, cytogenetic, and molecular genetic evaluation, the final sex assignment was male in 47 (48.0%) patients and female in 41 (41.8%) patients, while sex assignment was revised in 9 (9.2%) patients (Table 1). The distribution of cytogenetic categories is presented in Table 2, with 46,XY DSD being the most common diagnosis (37; 35.6%), followed by 46,XX DSD (30; 28.8%) and sex chromosome DSD (21; 20.2%). Culture failure occurred in 10 (9.6%) cases.

**Table 1:** Demographic and clinical characteristics of patients with disorders of sex development Values are presented as mean ± standard deviation (SD) or number (percentage). Percentages are calculated using the total study population (N = 98).

Characteristic	Number of Cases, n (%)
Total patients, N	98
Age (years), mean ± SD	13.5 ± 12.3
Age at presentation, n (%)	
Birth to 1 month	8 (8.2)
1 month to 1 year	6 (6.1)
1 to 5 years	12 (12.2)
>5 years	62 (63.3)
Sex assigned at referral, n (%)	
Male	54 (55.1)
Female	44 (44.9)
Male:Female ratio	1.2:1
Final sex assignment after evaluation, n (%)	
Male	47 (48.0)
Female	41 (41.8)
Sex reassignment following evaluation	9 (9.2)

**Table 2:** Cytogenetic classification of patients with disorders of sex development SCDSD: sex chromosome disorders of sex development.

Chromosomal Abnormality	Number of Cases, n (%)
46,XY	37 (35.6)
46,XX	30 (28.8)
Sex chromosome DSD (SCDSD)	21 (20.2)
Culture failure	10 (9.6)
Total	98 (100)

Consanguinity was observed in one-third of the families (33 (33.7%)), reflecting a trend common across various Indian communities. Patients were approximately evenly distributed between urban and rural areas, with varying levels of awareness and access to referral services. A family history suggestive of DSD was present in 18 (18.3%) patients, with most cases related to endocrine issues. This indicated a possible genetic association in a particular subset.

Cytogenetic findings

Culture failure was observed in 10 (10.2%) samples. Culture failure indicates unsuccessful lymphocyte growth, resulting in inadequate metaphase spreads for chromosomal analysis despite repeated culture attempts. These patients were retained in the cohort because they were referred with clinically suspected DSD and contributed to the overall laboratory diagnostic workload.

Clinical correlations

Androgen exposure was the main finding in the 46,XX DSD group (Table 2). Clinical presentations included penoscrotal hypospadias and ambiguous genitalia. Developmental delays or syndromic associations were present in 42 (42.9%) cases. This shows that the cases involved more than genital differences alone.

Characteristics of patients with 46,XY DSD (Tables 3, 4) ranged from severe undervirilisation or cloacal anomalies to a typical male appearance, often associated with concerns about fertility or hernias. In 18.9% of cases, an inguinal hernia was the first symptom. This suggests that even minor signs in younger patients should prompt karyotyping.

**Table 3:** Clinical presentation according to cytogenetic category: 46,XX DSD cases DSD: disorders of sex development.

Clinical Feature	Number of Cases, n (%)
Penoscrotal hypospadias with internal female structures	10 (33.3%)
Ambiguous genitalia	7 (23.3%)
Short stature	5 (16.7%)
Cloacal exstrophy	4 (13.3%)
Ovarian dysgenesis	4 (13.3%)
Total	30 (100%)

**Table 4:** Clinical presentation according to cytogenetic category: 46,XY DSD cases DSD: disorders of sex development.

Clinical Feature	Number of Cases, n (%)
Short stature	7 (18.9%)
Ambiguous genitalia	8 (21.6%)
Inguinal hernia	13 (35.1%)
Hypospadias	5 (13.5%)
Cloacal exstrophy	4 (10.8%)
Total	37 (100%)

Five adolescents showed classic features of Turner syndrome, including short stature and delayed puberty. These typical features led to further assessment. Phenotypes ranging from undervirilised males to Turner-like characteristics were observed in patients with mosaic variants such as 45,XO/46,XY. These phenotypes highlight mosaic distribution and tissue-specific expression (Table 5).

**Table 5:** Distribution of sex chromosome disorders of sex development

Chromosomal Abnormality	Number of Cases, n (%)
45,X (Turner syndrome)	5 (23.8%)
Mosaic 45,X/46,XY (mixed gonadal dysgenesis)	4 (19.0%)
Mosaic 45,X/46,XX	3 (14.3%)
47,XXY (Klinefelter syndrome)	3 (14.3%)
46,XX/47,XXX	2 (9.5%)
47,XXX	2 (9.5%)
46,XY/45,X	2 (9.5%)
Total	21 (100%)

Advanced genetics and diagnostic yield

In over one-fifth (n = 22, 22.1%) of the cases, further work-up was needed. Most of these cases (n = 12) were confirmed using FISH. CMA identified clinically meaningful CNVs in half of the selected samples (n = 2). WGS, although performed in only three highly complex cases, provided definitive diagnoses in all of them. A group of representative cases demonstrates the complementary diagnostic utility of Sanger sequencing, CMA, and WGS in clinically challenging DSD cases (Table 6).

**Table 6:** Representative cases illustrating the diagnostic utility of advanced genetic investigations in disorders of sex development DSD, disorders of sex development; CMA, chromosomal microarray analysis; WGS, whole-genome sequencing; MRI, magnetic resonance imaging; CAH, congenital adrenal hyperplasia; BMP, bone morphogenetic protein.

Case	Age/Sex	Clinical Presentation	Cytogenetic Finding	Advanced Genetic Investigation	Final Diagnosis/Key Findings	Clinical Impact
1	27-year-old, male	Penoscrotal hypospadias, bilateral undescended gonads, phallus with hypertrophied clitoris; MRI demonstrated uterus and ovaries	46,XX	Sanger sequencing	Compound heterozygous pathogenic CYP21A2 variants (c.293-13C/A>G (I2G) and c.1069C>T (p.Arg357Trp)), consistent with 21-hydroxylase deficiency (46,XX DSD due to congenital adrenal hyperplasia)	Confirmed molecular diagnosis, facilitated informed gender-affirming management, and guided total abdominal hysterectomy with bilateral salpingo-oophorectomy followed by staged hypospadias repair while respecting the patient's male gender identity.
2	8-month-old, male	Severe growth failure, complete cleft lip and palate, bilateral optic disc coloboma, global developmental delay, multiple congenital anomalies	46,XY,der(15)t(10;15) (p11.1;p11.1), mat	Chromosomal microarray analysis (CMA)	41.7 Mb duplication of chromosome 10p15.3–q11.21 and 1.3 Mb deletion of chromosome 3p26.3 involving CRBN and TRNT1; maternal inheritance confirmed	Demonstrated the complementary role of CMA in defining pathogenic copy number variants and fully characterising a complex chromosomal rearrangement identified by conventional cytogenetics.
3	Four phenotypic females (14–25 years)	Primary amenorrhoea with varying degrees of virilisation; one patient with androgen insensitivity syndrome following bilateral gonadectomy, others with inguinal testes, hypoplastic uterus, absent ovaries, or clitoromegaly	46,XY	Hormonal evaluation, imaging and targeted molecular genetic investigations	Spectrum of 46,XY DSD with variable phenotypic expression, including androgen insensitivity syndrome and disorders of gonadal development	Highlighted the marked phenotypic heterogeneity of 46,XY DSD and underscored the importance of integrating clinical assessment, endocrine evaluation, imaging and molecular testing for accurate diagnosis and management.
4	4-year-old, male	Bilateral undescended testes, craniofacial dysmorphism, limb deformities, developmental delay, and multiple congenital anomalies	46,XY,t(15;Y) with mosaicism	Whole-genome sequencing (WGS)	Multiple clinically relevant structural variants affecting genes involved in BMP signalling and skeletal/craniofacial development; among 48,150 structural variants, 29 clinically significant variants were prioritised	Demonstrated the added diagnostic value of WGS in identifying complex structural genomic alterations beyond conventional cytogenetics, improving genotype–phenotype correlation in syndromic DSD.

Representative cases

A 27-year-old male was observed to have penoscrotal hypospadias and undescended gonads on examination in the urology outpatient department. A phallus with a hypertrophied crus of the clitoris was observed, which raised concerns about a possible DSD. MRI of the abdomen revealed internal female genital organs, including ovaries and a uterus-like structure. Karyotyping performed at our institute revealed a 46,XX karyotype in 20 metaphase spreads, consistent with female sex chromosomes. Further genetic evaluation using Sanger sequencing revealed compound heterozygous pathogenic mutations in the CYP21A2 gene, known to be associated with 21-hydroxylase deficiency. The patient was compound heterozygous positive for the CYP21A2:c.293 13C/A>G(I2G) and c.1069>T(R356W) hotspot mutations. The patient decided to maintain his social identity as male, and therefore, a total abdominal hysterectomy and bilateral salpingo-oophorectomy were performed, followed by a two-step hypospadias repair with successful restoration of the penile urethra.

An eight-month-old male infant presented with severe growth failure, multiple congenital anomalies, including a complete cleft lip and palate and bilateral optic disc coloboma, and global developmental delay. Initial karyotyping revealed 46,XY,der(15),t(10;15)(p11.1;p11.1),mat. CMA identified a 41.7 Mb duplication at 10p15.3-q11.21 encompassing 140 OMIM genes, including GATA3, and a 1.3 Mb deletion at 3p26.3 involving the CRBN and TRNT1 genes. Parental analysis confirmed maternal inheritance of both abnormalities, indicating a complex chromosomal rearrangement with clinical consequences for the proband. This case demonstrated the utility of integrating conventional cytogenetics with high-resolution array analysis to fully characterise complex chromosomal abnormalities and their clinical implications.

Four female patients (aged 14-25 years) presented with primary amenorrhoea and various degrees of virilisation. All of them identified as female and had a 46,XY karyotype, with varying clinical presentations. A 14-year-old patient with known androgen insensitivity syndrome and a history of bilateral gonadectomy showed germ cell neoplasia on histopathology. Hirsutism, late menarche, and bilateral testes in the inguinal region were observed in a 15-year-old. Another 19-year-old presented with primary amenorrhoea, a hypoplastic uterus, and absent ovaries, while a 25-year-old presented with primary amenorrhoea, clitoromegaly, and absent ovarian development. Imaging, hormone tests, and genetic analysis were needed for a thorough evaluation.

A four-year-old boy with DSD had multiple anomalies, including bilateral undescended testes, limb deformities, and craniofacial dysmorphism. He also had developmental delays and other birth defects. The karyotype showed 46,XY,t(15;Y) with mosaicism. Structural changes affecting BMP signalling and harmful mutations in several genes linked to skeletal and craniofacial development were identified through whole-genome sequencing of the patient and his parents. Among a total of 48,150 SVs, 29 were clinically correlated and selected for further study. Of the 29 SVs, 14 were heterozygous, and 15 were homozygous. A total of 18 deletions, eight translocations, two insertions, and one duplication were observed. Later, the genes involved were checked for clinical correlations using several gene databases. 

## Discussion

The practical approach to DSD diagnosis in Eastern India was demonstrated through three years of experience. Combining traditional cytogenetics with modern genomic technologies was highlighted as improving diagnostic accuracy. 

Compared with various national and international datasets (Table 7), the most common chromosomal pattern observed in this study was 46,XY DSD [19-24]. Our findings align with reports from previous Indian studies, including that of Misgar et al. (2019) in Kashmir. Misgar et al. reported equal representation of 46,XX and 46,XY DSD [20]. Variations in genetics, environmental factors, and referral practices suggest regional differences. 

**Table 7:** Comparison of DSD category prevalence across studies DSD, disorders of sex development, SCDSD: sex chromosome disorders of sex development.

Study	Location	Study Period	No. of Cases	46,XY DSD, n (%)	46,XX DSD, n (%)	SCDSD, n (%)
Current Study	Eastern India	2022–2024	98	37 (35.6%)	30 (28.8%)	21 (20.2%)
Man et al. 2022 [19]	UK	1995–2019	607	371 (61.1%)	168 (27.7%)	68 (11.2%)
Misgar et al. 2019 [20]	Kashmir Valley, India	2008–2018	73	29 (39.7%)	29 (39.7%)	15 (20.5%)
Kohva et al. 2018 [21]	Finland	2004–2014	550	293 (53.3%)	53 (9.6%)	204 (37.1%)
Mazen et al. 2018 [22]	Egypt	2013–2014	108	–	–	48 (44.4%)
Ganie et al. 2017 [23]	South Africa	1995–2014	416	239 (57.5%)	137 (33.0%)	40 (9.5%)
De Paula et al. 2016 [24]	Brazil	1989–2011	408	250 (61.3%)	124 (30.4%)	34 (8.3%)

In our data, the percentage of sex chromosome abnormalities, including Turner syndrome and its mosaic forms, was higher than that in many global studies [19,23,24]. Children with specific phenotypic signs, such as short stature, inguinal hernias, or delayed puberty, are referred more quickly for cytogenetic evaluation at our institution. This may explain the higher percentage observed. Additionally, this suggests that a robust cytogenetics service is necessary in areas where multidisciplinary care for DSD is being developed [5]. 

The 46,XY DSD group exhibited pronounced phenotypic variability. Since over half of the 46,XY DSD cases remain undiagnosed, even minor genital differences or isolated issues, such as an inguinal hernia in infants, should not be overlooked. Unnecessary psychological stress for families can be avoided through early cytogenetic testing [25].

Representative case analysis

Divergence in gender identity presented male phenotype with 46,XX karyotype caused by a mutation in the CYP21A2 gene with congenital adrenal hyperplasia, which is a hereditary metabolic disorder where overexposure of the androgen hormone in a female fetus throughout life is the cause of the disease [7,26]. This case also highlights the importance of early diagnosis with hormonal therapy, a multidisciplinary approach for sexual reassignment surgery and decision-making for gender identity and genetic counselling to avoid psycho-social stigma [3].

The 10p deletion case shows multiple CNVs and complex chromosomal rearrangements. A maternal balanced translocation was observed in the CMA reports, which explained the intricate traits exhibited by the genetically unbalanced offspring. Recognising how both increases and decreases in genetic material affect growth helps to understand gene dosage [27,28]. 

The need for ongoing monitoring of hormonal regulation and cancer risk is shown in female patients with a 46,XY genotype, which also reveals a range of phenotypes within one chromosomal category. Similar to the reports of previous literature, which reports a higher cancer risk linked with dysgenetic gonads [3,25], the presence of germ cell neoplasia was found in the gonadectomy specimen of one of the 46,XY DSD patients who was being reared as a female. 

The model for the future of DSD genetics, illustrated by the case of a 4-year-old boy with DSD and multiple anomalies, incorporates WGS and highlights its use in assessing complex syndromic features and outcomes. Identifying structural variations that influence developmental pathways helps in understanding the mechanisms that can inform treatment options [5,29].

The pattern of chromosomal distribution aligns with much of the Indian literature [7,20]. However, it does not align with Western studies [19,21-24]. This shows that population-specific factors influence DSD patterns.

The diagnostic yield indicates the effectiveness of current procedures across various healthcare settings and is consistent with global experience. This demonstrates that, even in resource-limited environments, the successful application of these technologies enables comprehensive genetic screening [17].

Clinical implications and future directions

The current study warrants incorporating modern genetic tools into traditional DSD screening procedures because of the high diagnostic yield. Based on the clinical presentation, CMA, FISH, and WGS can be strategically used to enhance diagnostic efficiency and manage costs. The discovery of new genetic variants and chromosomal patterns enhances the understanding of DSD genetics globally. This knowledge may reveal population-specific mechanisms that necessitate unique management strategies [30].

Study limitations

As the study involved retrospective selection, bias could occur. This is more likely in cases involving advanced genetic testing. The short follow-up period limits the assessment of long-term results and cancer risks. The larger DSD patient population is not accurately represented by this single-centre approach. Due to financial constraints, advanced techniques were not used in a larger number of patients.

## Conclusions

This study summarises the genetic findings and clinical presentation of DSD patients evaluated at a tertiary centre in Eastern India over a three-year period. The use of advanced diagnostic techniques, such as FISH, CMA, and WGS, in conjunction with traditional cytogenetics, has significantly enhanced the ability to reach a clear diagnosis, particularly in complex and ambiguous cases. These findings demonstrate that a systematic, genetics-focused approach facilitates more informed decisions regarding gender assignment, ongoing monitoring, and family counselling. By providing relevant local data, this research adds to the growing Indian literature on DSD and highlights the need for accessible molecular diagnostic services. 
